# A Case to Help

**Published:** 1888-11-24

**Authors:** 


### A CASE FOR HELP.

Lady Rothschild has sent a donation of £3, and Miss Spencer, of Bath, one of 10*s.*, on behalf of Charles Bridgett. It will be remembered that Bridgett is a consumptive patient, whose case the physician in attendance thinks may be permanently benefited by a prolonged stay at the Ventnor Hospital. The whole sum needed for his expenses, and to pay the weekly charges of the hospital, is £7. Of this, £3 10*s.* 0*d.*, as above stated, has already been contributed. If some of our readers will make up the remainder, Bridgett can be sent at once to Ventnor before the dangerous weather of winter comes on. Donations may be forwarded to Mrs. Young, matron of the Ironmongers' Almshouses, Kingsland Road, E.

